# Advances in Tumor Screening, Imaging, and Avatar Technologies for High-Grade Serous Ovarian Cancer

**DOI:** 10.3389/fonc.2014.00322

**Published:** 2014-11-18

**Authors:** Anders W. Ohman, Noor Hasan, Daniela M. Dinulescu

**Affiliations:** ^1^Division of Women’s and Perinatal Pathology, Department of Pathology, Eugene Braunwald Research Center, Brigham and Women’s Hospital, Harvard Medical School, Boston, MA, USA

**Keywords:** fallopian tubal secretory epithelial cell, high-grade serous carcinoma, genetically engineered mouse models, avatar models, serous tubal intraepithelial carcinoma, near-infrared fluorescence, the Cancer Genome Atlas, BRCA

## Abstract

The majority of high-grade serous ovarian carcinoma cases are detected in advanced stages when treatment options are limited. Surgery is less effective at eradicating the disease when it is widespread, resulting in high rates of disease relapse and chemoresistance. Current screening techniques are ineffective for early tumor detection and consequently, *BRCA* mutations carriers, with an increased risk for developing high-grade serous ovarian cancer, elect to undergo risk-reducing surgery. While prophylactic surgery is associated with a significant reduction in the risk of cancer development, it also results in surgical menopause and significant adverse side effects. The development of efficient early-stage screening protocols and imaging technologies is critical to improving the outcome and quality of life for current patients and women at increased risk. In addition, more accurate animal models are necessary in order to provide relevant *in vivo* testing systems and advance our understanding of the disease origin and progression. Moreover, both genetically engineered and tumor xenograft animal models enable the preclinical testing of novel imaging techniques and molecularly targeted therapies as they become available. Recent advances in xenograft technologies have made possible the creation of avatar mice, personalized tumorgrafts, which can be used as therapy testing surrogates for individual patients prior to or during treatment. High-grade serous ovarian cancer may be an ideal candidate for use with avatar models based on key characteristics of the tumorgraft platform. This review explores multiple strategies, including novel imaging and screening technologies in both patients and animal models, aimed at detecting cancer in the early-stages and improving the disease prognosis.

## Treatment Approaches for Patients and High-Risk Women

Ovarian cancer is the most lethal gynecological malignancy, with the majority of high-grade serous carcinoma (HGSC) cases being diagnosed in late stages of disease [for a comprehensive review see Bast (1)]. The current American Cancer Society statistics estimates that the 5-year survival is 44% when all disease stages are included but declines to 25% if only advanced stage cases are considered (1–3). Initial treatment for HGSC patients involves debulking surgery, which typically includes a combination of hysterectomy, bilateral salpingo-oophorectomy, and removal of the omentum, followed by therapy using platinum compounds and taxanes (4, 5). While platinum-based chemotherapy improves patient survival, the treatment is also very toxic; in addition, disease relapse following treatment and the acquisition of platinum chemoresistance are frequent events, suggesting a need for alternative treatment modalities (6). One strategy involves the addition of bevacizumab, a monoclonal antibody targeting VEGF-A, which has been tested together with standard chemotherapy in the setting of platinum-resistant recurrent disease. Indeed, the combination of an anti-angiogenic compound and single-agent chemotherapy improved objective response rates and progression free survival in comparison with chemotherapy alone, but the overall survival trend was not significant (7). An alternative strategy, which is aimed at reducing both the treatment toxicity and recurrence rates, proposes the administration of lower doses of carboplatin plus paclitaxel given once a week for 18 weeks instead of standard doses administered every 3 weeks for six cycles. Interestingly, this modified weekly regimen of reduced chemotherapeutic doses has recently been found to not only be an effective option for first-line treatment but, most importantly, to be associated with an enhanced quality of life as assessed physically, socially, emotionally, and functionally (8).

New treatment approaches could be beneficial not only for patients but also for women at increased risk for developing the disease. A recent study, which included a broadly spanning, exome-wide analysis of both ovarian cancer somatic and germline mutations, has estimated that more than 20% of women likely have an inherited predisposition to ovarian cancer (9). The most studied mutations involve the *BRCA* family of tumor suppressor genes, which confer an increased risk for developing breast and ovarian cancers (10). The lifetime risk for developing breast cancer in *BRCA* mutation carriers varies between 56 and 84% while the ovarian cancer risk ranges from 36–46% to 10–27% for women with *BRCA1* and *BRCA2* mutations, respectively (11). In addition, lifetime risks for both breast and ovarian cancers due to *BRCA* mutations appear to have increased over time, possibly due to lower physical activity and higher BMIs (12). When assessing individual patient risks for ovarian cancer it is important to note that irrespective of genetic risk, women with irregular menstrual cycles experience a 2.4 times greater incidence of death due to ovarian cancer (13).

## Quality of Life after Prophylactic Surgery in High-Risk Women

The mean age at the time of diagnosis for the average population is 63 years; by comparison, the mean age for BRCA mutation carriers is considerably lower at 50.8 years. For both groups, the cancer risk increases with age, especially after menopause. Consequently, the current recommendation for *BRCA* carriers is to undergo risk-reducing surgery once childbearing is completed, since they tend to be diagnosed at an earlier age than sporadic ovarian cancer (14). Standard prophylactic surgical options include bilateral mastectomy and salpingo-oophorectomy, which confer the most substantial reduction in cancer risk and increase in life expectancy (10, 15, 16). Studies looking at the efficacy of bilateral prophylactic salpingo-oophorectomy in *BRCA* mutation carriers concluded that it not only resulted in a 96% reduction in the risk of developing coelomic epithelial cancers but also decreased the breast cancer risk by 53% in comparison with *BRCA* mutation carriers who chose not to undergo the procedure (14). Despite the drastic risk-reduction associated with prophylactic surgery, it is far from an ideal treatment approach. In addition to being stripped of their inherently female characteristics, women who choose to undergo a combination of mastectomy, bilateral salpingo-oophorectomy, and hysterectomy are faced with a significant decrease in their quality of life, premature menopause, hot flashes, decreased cognition and sexual function, and increased risk of osteoporosis and cardiac mortality (14, 17–19). The use of hormone therapy and other medications may mitigate some of these adverse effects. Nevertheless, hormone replacement therapy is controversial, especially in high-risk women, as it has been linked to an increased risk of breast cancer (20). While an elective oophorectomy procedure may correlate negatively with life expectancy in women at average risk, the procedure remains beneficial for women at increased risk (21). Despite the possible negative physiological and psychological impacts of prophylactic oophorectomy, women at increased risk for developing HGSC report less anxiety about developing the disease, which they believe compensates for undesirable side effects (16). The primary cause of depression reported amongst post-surgical women is due to sexual dysfunction (21). In addition, *BRCA* mutation carriers may also experience the fear of transmitting a hereditary disease to their children. Women with these reproductive concerns could choose to investigate alternative methods, including *in vitro* fertilization and screening of embryos via preimplantation genetic diagnosis in order to eliminate the chance of transmitting faulty *BRCA* genes to their children (17).

## Non-Surgical Risk-Reduction Approaches in *BRCA* Mutation Carriers

There are limited risk-reducing approaches for women with *BRCA* mutations who choose not to undergo prophylactic surgery (22). Current strategies include early breast cancer screening consisting of annual mammogram and breast MRI. In addition, gynecologic cancer screening consists of baseline transvaginal ultrasound and CA-125 serum level measurements followed by frequent monitoring using ROCA evaluation protocols. Breast cancer screening results in early tumor detection and a survival advantage; in contrast, ovarian cancer screening has yet to be associated with a significant reduction in mortality (23). For example, 63% of ovarian cancers detected were stage IIC or higher in a comprehensive study of over 6000 high-risk women (23). This can be partially attributed to the fact that current screening options are limited and not best suited to detect early-stage cancers (24). An alternative strategy involves the use of chemopreventive methods, including selective estrogen receptor inhibitors, tamoxifen and raloxifene, in addition to oral contraceptives (15). Oral contraceptives have indeed been shown to decrease the risk of developing ovarian cancer in the general population (25). However, the use of oral contraceptives in *BRCA* mutation carriers, while beneficial for ovarian cancer prevention, may be associated with an increased risk for developing breast cancers (26, 27).

## Development of Imaging Technologies Aimed at Early Detection and Improving Patient Outcome

As mentioned above, the vast majority of both familial and spontaneous HGSC cases are diagnosed in advanced stages, at which point the prognosis is poor (28). Because there are few clear early symptoms, developing effective methods for early detection is paramount to improving long-term patient survival (29). Current methods of detection include non-invasive screenings using serum CA-125, ultrasound, sonography, CT, and MRI scans (30, 31). A laparoscopic procedure may also be used to provide an image of the lower abdominal organs, as well as attain a biopsy, which is necessary in order to confirm cancer diagnosis. These tools can give information regarding the size, composition, and location of the tumor and whether it has spread, which will be important for disease staging and treatment plans (28, 32). In terms of early detection, it is important to note that current imaging technologies are not able to distinguish precursor lesions inside the fallopian tube or ovary, two of the tumor initiation sites for HGSC. Novel *in vivo* imaging devices, such as confocal microlaparoscopes, which are instrumental in providing live images of abnormal regions and guiding biopsies, may be better equipped to assist with early tumor diagnosis (33). Images obtained by these probes in real-time during surgery have shown a clear distinction between normal and abnormal regions within the ovarian surface epithelium (33). Furthermore, studies using a flexible microlaparoscope have demonstrated the ability to provide high-resolution images of early stage cancer inside the fallopian tube in the intra-operative setting, thereby facilitating both an earlier diagnosis and accurate disease staging (34). Since these procedures have shown merit in surgical settings, they may become a viable complementary option to traditional biopsies as future modalities of disease confirmation (33). Nevertheless, the specificity and sensitivity of this method have yet to be determined in clinical trials and there are also drawbacks associated with this procedure. For example, the image quality produced by the microlaparoscope is diminished when compared to traditional laparoscopes due to a smaller scope size, focal distance, and a reduced light output. Procedural complications may also arise from a lack of tight correlation between the movement of the instrument handle and instrument movement in the surgical field due to its increased flexibility (35). Other considerations include a reduced imaging depth, which may limit the ability to view cells below the tissue surface layer, as well as the development of non-toxic and non-mutagenic contrast-enhancing agents (33). Nevertheless, the device can successfully image organs *in vivo* without major complications (33) and needs to be carefully evaluated in future clinical trials.

Another widely used primary diagnostic tool for women seeking evaluation of a pelvic mass is transvaginal sonography (TVS). Traditional TVS technologies are being replaced by contrast enhanced transvaginal sonography (CE-TVS) and transvaginal color Doppler sonography (TV-CDS), which can better depict the tumor morphology by analyzing its microvasculature (31). In a study aimed at evaluating the diagnostic ability of contrast enhanced 3-dimensional power Doppler sonography (CE-3D) relative to conventional 3D Doppler sonography, the CE-3D technology showed 95.6% accuracy in distinguishing between benign and cancerous tumors compared to 86.7% accuracy for the conventional method (36). Contrast-enhancing agents coupled with transvaginal and Doppler sonography may merit further investigation as an early screening tool for women at risk. It is worth mentioning that the development of selective means for tumor imaging is critical not only for early detection but also for improving patient outcome (29). Thus, the use of selective intraoperative tumor imaging devices has the potential to both improve disease staging and enhance precision during cytoreductive surgery as it allows for better visualization of tumors (37). For example, a combination of functional *in vivo* and anatomical *ex vivo* X-ray micro-computed tomography (μCT) can provide a highly detailed three-dimensional analysis of the tumor micromorphology, vascularization, and accurately quantify relative blood volumes (rBV) in tumors, which can further inform treatment plans (38). Most importantly, the study has found a direct correlation between microvascular parameters (i.e., vessel size, the complexity of vessel branching) and tumor angiogenesis and aggressiveness (38).

Further advances have been made in ultrasound technology as well. Photoacoustic imaging is an emerging technology based on the photoacoustic effect, which is generated when tissues are pulsed with non-ionizing lasers. This results in a transient thermoelastic expansion and emission of an acoustic wave, which is detected by ultrasonic transducers and converted into images (39). A study evaluating biodegradable photoacoustic imaging agents in animal models of ovarian cancer found that cellulose nanoparticles produced high contrast signals. Interestingly, cellulose nanoparticles demonstrated a significant increase in signal when compared to gold nanoparticles, which are commonly used in photoacoustic imaging. Unfortunately, this imaging agent only proved to be biodegradable *ex vivo*. For the purpose of reducing toxicity and to facilitate clinical translation, further research will need to focus on nanoparticles that biodegrade within the mammalian circulatory system (40).

A strong emphasis is currently placed on evaluating imaging agents that are safe to use in patients and allow the visualization of early or recurrent tumors. Current research in ovarian cancer investigates the use of the folate receptor α (FR-α) and HER-2 as targeted agents for tumor-specific fluorescence imaging. Thus, FR-α is overexpressed in the majority of epithelial ovarian cancers, especially in HGSC tumors with a high risk of recurrence. Increased FR-α expression is detected not only in primary tumors but also in metastatic foci and recurrent tumors (41). Most importantly, chemotherapy does not appear to significantly alter FR-α expression in patient tumors (37, 41). In addition, FR-α-targeted fluorescent agents are able to selectively enhance imaging of tumor cells (37). PPF, an FR-targeted probe that is well suited for both PET and optical imaging was investigated *in vivo* in a trial using primary cell xenografts, *in vitro* with primary human ovarian cancer cells, and *ex vivo* with omentum removed from xenografts (42). PPF injected either intraperitoneally or intravenously was able to identify FR-positive primary HGSC tumors and their metastases to the omentum. As FR is overexpressed in HGSC, FR-targeted probes, such as PPF, may bear great utility in the clinical setting. This method could be ideal for guiding surgery due to the non-invasive, high-resolution, real-time images it produces (42). The unique features of this novel imaging tool may prove useful for ovarian cancer detection and monitoring. Furthermore, fluorescence imaging using FR-α-targeted agents could play a critical role during debulking surgery, as current methods of imaging are not tumor-specific.

Fluorescence imaging has been shown to detect a greater number of tumors when compared to conventional methods. Interestingly, a vinblastine folate-targeted drug, vintafolide, when used in combination with a diagnostic imaging tool, etarfolatide, may merit attention as a means to advance personalized treatments. Thus, etarfolatide imaging has been used successfully to select patients with FR-positive platinum-resistant ovarian tumors who may benefit from folate-targeted therapy (43). This novel combination of folate-targeted agents for imaging and treatment resulted in a marked increase in progression free survival for platinum-resistant patients. Based on the highly selective nature of this treatment, the drug efficacy was reported to be greater and its toxicity decreased compared to standard therapy (43). An alternative strategy for selective tumor imaging involves the use of the HER-2 biomarker, which is overexpressed in advanced HGSC cases (44). Imaging using HER-2-targeted magnetic iron oxide nanoparticles allows both magnetic resonance and optical imaging of peritoneal tumors when used in orthotopic ovarian xenograft models (45). This technology, which enables tumor imaging with high specificity and resolution, can be instrumental in drug delivery and image-guided surgery (44, 45). A third strategy involves the development of near-infrared fluorescence (NIRF) imaging technologies, which have been successfully tested *in vivo* in both pancreatic and ovarian cancer models as an alternative to ultrasound, CT, and MRI scans (46). Gene expression profiling was instrumental in identifying proteases relevant to tumors, which further enabled the development of protease-specific NIRF probes. Such probes can provide not only a higher resolution for molecular-guided detection of early tumors but also the ability to distinguish between inflammation and cancer (46). Similarly, an alpha(v)beta(3-) integrin targeted NIRF probe was used successfully in ovarian xenograft models to optimize debulking surgery (47). Furthermore, the increased target to background ratio, high sensitivity (95%), specificity (88%), and diagnostic accuracy (96.5%) of this imaging system suggest that the NIRF-targeted platform is well suited for clinical translation and may be able to provide highly accurate images of small tumor lesions that are otherwise difficult to detect (47).

Plasma tumor biomarkers for the detection of early tumors and precursor lesions have been difficult to identify. CA-125 is presently used to help diagnose ovarian cancer, mainly for late stage cases, and to predict the chance of tumor recurrence (48). Nevertheless, CA-125 is not always a reliable biomarker for early stage tumors due to its lack of sensitivity (48). Large screening studies are currently underway to determine the sensitivity and specificity of a combined monitoring protocol using serum CA-125 levels and TVS for early diagnosis. The screening is based on an improved algorithm designed by Dr. Steven Skates to identify cancer risk based on rising trends in individual CA-125 levels (1). An alternative strategy involves the combined evaluation of multiple biomarkers in addition to CA-125. Research suggests that the addition of HE4, leptin, prolactin, osteopontin, insulin-like growth factor-II, CEA, and soluble vCAM cancer biomarkers to CA-125 serum surveillance protocols may result in a better diagnostic reliability when compared to CA-125 alone (1, 25). It is worth noting that recent studies suggest that a significant proportion of HGSC tumors originate from precursor lesions [serous tubal intraepithelial carcinoma (STIC)] located within the fallopian tube rather than the ovary, and this is the case especially in *BRCA* women (1, 49). Consequently, the development of serum screening tests and methods of diagnostic imaging need to include markers characteristic for the fallopian tube/fimbria in addition to the ovary. Currently, small early tumors within the fallopian tube cannot be detected via ultrasound or by measuring serum CA-125 levels (50). Comprehensive screening of such lesions through endometrial cytological testing may be a promising method for the early detection of HGSC in high-risk women and BRCA mutation carriers. Otsuka et al. reported that tumor cells shed from tubal STICs could be detected through careful examination of endometrial cytological samples (50). This would enhance the early detection rates for HGSC tumors as the occurrence of false positives through cytological testing appears to be low (50). Interestingly, this pilot study was able to detect malignant cells in five patients for whom imaging results were normal; three of them presented with no symptoms and were later diagnosed with early-stage HGSCs (50). In addition to further confirming the tubal site of origin for HGSC cases, the study also reported a 4-fold increase in the number of high-grade serous tumors being detected using this method when compared to other ovarian cancer subtypes (50). In contrast, the direct testing of cervicovaginal cytological samples yielded positive results in only one of five patients, suggesting that this is not an efficient means of detecting early-stage HGSC tumors.

## Generation of Improved Animal Models that Closely Resemble HGSC

In addition to aiding in the development of new imaging techniques, murine models of ovarian cancer are integral to drug development and disease pathogenesis studies (46, 51). While several genetically engineered mouse models (GEMM) have been previously developed for endometrioid ovarian cancer (52–54), clinically relevant models for HGSC have been difficult to generate. This could be due to model designs based solely on a traditional view of disease pathogenesis, such as the ovarian origin hypothesis. New clinical protocols, which involve the sequential sectioning and examination of the fimbrial (SEE-FIM) end of the fallopian tube pioneered by Dr. Christopher Crum, have identified precursor lesions for HGSC arising in secretory cells of the fallopian tube, namely p53 signatures and STICs (55–58). In addition, several groups have been instrumental in leading efforts for refining murine HGSC models. Recently, the first genetic model of *de novo* HGSC originating in fallopian tubal secretory epithelial cells (FTSEC) has been generated, which recapitulates key genetic alterations (*BRCA, TP53*, and *PTEN*) and precursor lesions (STICs) that are hallmarks of the human disease (59). In addition to offering mechanistic insight into the origin and pathogenesis of HGSC, this model provides a platform to explore tumor sensitivity to novel therapeutic agents and diagnostic imaging methods that include the distal fallopian tube in addition to the ovary (59). Recently, a second genetically engineered model of HGSC was described using the Ovgp-1 promoter to target SV40 large T-antigen-induced tumorigenesis in the fallopian tube (60). This model also displays neoplastic lesions of the fallopian tube that resemble human STICs and p53 signatures. The murine ovarian carcinomas have molecular characteristics that strongly resemble the human disease as well. Furthermore, gene expression analysis studies of Ovgp-1-driven tumors have identified a novel biomarker, topoisomerase II-alpha, which is overexpressed with mutant TP53 in both murine and human STICs and HGSCs but not in normal adjacent tissues (60). Most importantly, this model provides independent support for the hypothesis that HGSC may be primarily tubal in origin and mirrors the clinical progression of human HGSCs (61). The development of FTSEC-driven animal models will be instrumental in providing a platform to test newly emerging data from The Cancer Genome Atlas (TCGA) in an *in vivo* relevant system. The goal of the research community is to develop ovarian cancer models that recapitulate not only novel genetic/genomic alterations but also the histopathology and clinical behavior of HGSCs. Resolving the pathogenesis of HGSC and its precursor lesions will likely enable more efficient methods for early detection, tumor imaging, and cancer prevention. Furthermore, by using a combination of murine model studies and epidemiological data from patients, it will be important to determine if premenopausal women with *BRCA* mutations can be offered risk-reduction surgery in a multi-step procedure without undergoing surgical menopause and loss of fertility in their younger years.

As a complement to genetically engineered models, personalized patient-derived murine xenografts (“avatar mice”) have been developed, which are able to more accurately predict tumor responses to therapy. Xenograft tumor models have been used for decades to examine the behavior of various types of therapies within a living system (62). They can closely resemble the molecular and histological characteristics of the human cancer they are derived from (63, 64) and demonstrate clinical relevance by predicting the activity and effects of trial therapies (65). However, their predictive ability varies dramatically based on the cancer being studied, and multiple therapies, which tested well in xenografts, did not ultimately result in successful clinical trials (66–68). While traditional xenografts are created by generating and then engrafting established cell lines (51), a subcategory of xenografts produced by direct transfer of patient tumor tissue into immunocompromised mice (also known as explant xenografts or tumorgrafts) has mimicked the drug effects seen in humans much more closely than cell line xenografts (69). Furthermore, one study of personalized tumorgrafts involving a broad range of human-derived tumors and anticancer therapies demonstrated a positive clinical predictive value for drug resistance over 90% of the time (70). Unlike human cell cultures, which tend to result in increasingly homogenous populations with successive passages, direct tissue transplants more accurately represent the heterogeneous makeup and genetic diversity of the original tumor, including its relative cell proportions and overall genomic profile (71, 72). These tumor sections can be implanted orthotopically within the homologous source tissue in addition to being dispersed into the body cavity via intraperitoneal injection, as is the case for cell line xenografts (73, 74). Using these techniques, avatar models have been generated that closely recapitulate the tumor of a specific donor patient. This has led to the identification of personalized therapeutic regimens by creating a tailored stand-in for patient tumors prior to or alongside treatment (75). The use of tumorgraft testing surrogates, which are generated by using a specific patient’s own tissue, has increased over the last decade. This trend was initiated by a recent study that described the use of xenograft technologies to create personalized tumorgrafts for a total of 14 patients with a variety of cancer types (76). The study results identified optimal, non-obvious treatment choices with a high rate of clinical success (76). This process has been performed with similar success in models of lung (77), pancreatic (78), prostate (79), breast (80, 81), and fallopian tube (82) cancers. In addition to being highly representative of the morphology and progression of human cancers, it was found that the success of tumor engraftment is by itself a prognostic indicator of disease outcome for women with newly diagnosed breast cancer (80).

While GEMM and xenografts both strive to generate accurate models of human disease and their usage at times overlaps, they have individual features best suited to distinct roles in cancer research. Tumors that develop from a xenograft retain the natural genetic alterations derived from the original source (63, 83). Conversely, GEM models must recreate these changes based on the result of investigation or hypothesis, and therefore they often cannot replicate the complexity and genomic diversity found in patient tumors (74). In cancers with high variance in molecular alterations between patients, tumorgrafts should be used to test therapies in models that more accurately represent individual tumors (77), since generalized results from GEMM studies will not be applicable. Personalized tumorgrafts have also been used to identify changes in drug resistance at specific stages of disease by grafting repeatedly from the same patient at different time points (63). Furthermore, avatar models could also allow the preemptive identification of new treatment strategies necessary when a patient develops resistance to clinically available therapies (84). Being able to determine tumor sensitivity and drug resistance for each individual patient upfront would allow oncologists to attempt experimental treatments with a higher probability of success while retaining conventional therapies as an option (85). In contrast, GEMMs have attributes superior to avatar models when it comes to studying the origin of the disease, precursor lesions, tumor progression, and the contribution of the immune system to cancer pathogenesis by allowing the inducible targeting of key genes in a tissue-specific manner in immunocompetent mice (86). There are several challenges in creating avatar mice relative to GEM and cell line xenograft models, including the need for surgical extraction of adequate tumor samples from the patient, ideally including accessory tissue (72), and a high rate of implantation failure (85). While tumor heterogeneity is represented, the microenvironment is typically not. This drawback, combined with the use of immunocompromised mice, restricts how similarly avatar models behave when compared to human disease. Such limitations could be overcome by incorporating recent xenograft advancements. The tumor microenvironment can be retained in a xenograft by grafting stroma alongside the tumor (75) and the use of “humanized” models preserve immune system functionality after engraftment (87). Besides technical concerns, there is a high financial barrier for creating new avatar lines (75). However, once tissues have been extracted and implanted, human tumors can be serially passaged in mice, archived, and later repropagated from tumor banks (82). The generation of tumor banks enables the repeated testing and study of patient tumors from a small number of original extractions.

Further development of representative mouse models of HGSC is an urgent need for the field (88) and tumorgrafts have attributes suited to this subtype. HGSCs are characterized by rapid metastasis (89) and tumorgraft models were reported to metastasize to regions similar to those seen in patients (90). HGSCs are also characterized by genomic instability (51) and tumorgrafts were shown to accurately retain the genomic profile of the original patient sample in animal models throughout multiple grafts (72). Avatar mice have been generated for fallopian tube carcinoma (82), which supports their viability for ovarian cancer, particularly in conjunction with the tubal origin hypothesis (91). This year, the first large-scale tumorgraft mouse study of ovarian cancer was published, consisting of 168 engrafted models from patient samples, which were representative of the entire spectrum of the disease (83). As in previous tumorgraft studies, the models closely resembled the patients they were derived from. The majority of models that developed ascites originated from patients with ascites, which is notable as the development of ascites is characteristic of HGSC (89). Consistent with prior comparisons between cell line xenografts and donor patient platinum sensitivity in ovarian cancer (92), all of the ovarian models tested for platinum sensitivity had the same type of response as the donor patient (83). It has been suggested that the tumor microenvironment may play a role in the high rate of relapse and increased drug resistance seen in HGSC (93). These ovarian tumorgrafts strongly resemble the source microenvironment by inducing the formation of tumor stroma (83). As shown in a larger tumorgraft study (72), the ovarian models closely resembled the source patient’s genomic alterations after engraftment and the grafts implanted in clinically relevant sites. Finally, just as demonstrated previously (80), the initial success of a graft tended to correlate negatively with patient survival (83). This extensive study demonstrates the feasibility of ovarian tumorgrafts as patient surrogates, particularly given the reasonably accurate representation of the disease diversity (94). Consequently, the generation of avatar mouse models for HGSC is expected to assist oncologists with establishing individual resistance profiles quickly in a surrogate model following biopsy and informing patients of therapy choices in real-time (85).

Avatar tumorgrafts have been found to be highly predictive models clinically and the generation of such models can aid with drug design on an individual patient basis (67, 76). The use of avatar models alongside patients in concurrent clinical trials has already been proposed (75), though it is noted that variance in how accurately these strains reflect human disease, including the contribution of the immune system, can be a confounding factor. In recent years, there has been renewed interest in harnessing the immune system to target cancers (95, 96), often achieved by blocking the inhibitors of immune reactions elicited by tumor cells. This approach has led to considerable success, such as the use of ipilimumab immunotherapy in metastatic melanoma (96). Ipilimumab is a monoclonal antibody against CTLA-4 and promotes effective antitumor targeting by cytotoxic T lymphocytes. Therapies targeting CTLA-4 have also been investigated in ovarian cancer; a combined treatment, which involves blockade of CTLA-4 and PD-1 and boosts the immune system, was found to induce tumor rejection in 75% of tumor models examined (97). In addition, the endothelium of many tumors may function as a primary defense against immune system activation by creating a physical protective barrier for the tumor and resisting immune cell invasion (98). It remains to be seen whether the development of effective immunotherapies will increase the efficacy of conventional therapies and achieve durable remissions in patients (96). Notably, it was recently demonstrated that traditional therapeutic regimens could be modified to effectively recruit the immune system in the setting of platinum-resistant relapsed disease (6). While platinum and paclitaxel are often delivered at maximum tolerable doses, the dose-dense chemotherapy study demonstrates that delivery of conventional drugs in low doses within frequent intervals can enhance natural antitumor immune responses and reduce immunosuppression, thus leading to increased treatment efficacy (6). Thus, a dose-dense chemotherapy regimen was successful in promoting the antitumor CD8^+^ T-cell response in both mouse models and patients and reduced the tumor ability to suppress the immune system. This bodes well for further optimization of such treatments for individual needs, particularly as an extended weekly, dose-dense carboplatin and paclitaxel regimen has been shown to be effective in heavily pre-treated, recurrent, platinum-resistant ovarian cancer patients (99). Clearly, optimizing these treatments for patients requires a clinically translatable graft model. Xenograft models with an implanted functional human immune system have been previously developed to investigate viral and immune disease (100). Interestingly, such models were shown to be functional in cancer as well (87). A key benefit of GEMMs over xenografts has always been that they retain normal immune function (51). However, a xenograft with a humanized immune system, which is interacting with human tumor cells, may be more translatable to designing immunotherapies for patients based on individual needs. While GEMMs may ultimately prove more useful than avatar models for understanding the intricate details of HGSC origin and progression, personalized tumorgrafts will be key for the design of individual therapeutic regimens.

## Conclusions

Advances in tumor screening and imaging may help determine the optimal time to employ risk-reducing surgical approaches in women at high risk for HGSC, including *BRCA* mutation carriers. Prophylactic surgery offers the most significant reduction in the risk of developing breast and ovarian cancers, as current surveillance methods are not effective enough to lend support for ovarian conservation in premenopausal women at high risk. Bilateral salpingo-oophorectomy is, however, associated with multiple physiological and psychosocial side effects that may contribute to a decrease in the quality of life and a loss of fertility in younger women. Consequently, the use of endometrial cytological testing in high-risk women or improved *in vivo* imaging devices (i.e., confocal microlaparoscopes, photoacoustic imaging, μCT, and contrast enhanced 3D Doppler sonography) could prove to be more effective for both early detection and treatment. In addition, alternative strategies for tumor-specific imaging, which involve the use of FR-α-targeted fluorescent agents, HER-2-targeted magnetic iron oxide nanoparticles, or protease-specific NIRF probes, merit further investigation as selective tools for early tumor detection, monitoring, and image-guided surgery. It is worth noting that animal models are a valuable tool in ovarian cancer research for the purpose of developing and testing novel imaging technologies, biomarkers, and experimental treatments. However, there are currently a limited number of animal models available for HGSC. Advances in tumor xenograft technologies have enabled the development of personalized avatar mouse models, which have emerged as an ideal drug-testing platform, especially in concurrent clinical trials. Additionally, tumorgrafts appear to have qualities well suited to model HGSC. We suggest that avatars have the potential to improve patient outcome and quality of life by reducing the cost and toxicity of ineffective imaging and treatment.

## Author Contributions

Anders W. Ohman, Noor Hasan, and Daniela M. Dinulescu wrote the manuscript.

## Conflict of Interest Statement

The authors declare that the research was conducted in the absence of any commercial or financial relationships that could be construed as a potential conflict of interest.

## Abbreviations

FR-α, folate receptor α; FTSEC, fallopian tubal secretory epithelial cell; GEMM, genetically engineered mouse models; HGSC, high-grade serous carcinoma; NIRF, near-infrared fluorescence; STIC, serous tubal intraepithelial carcinoma; TCGA, the Cancer Genome Atlas.
